# Key chemokines direct migration of immune cells in solid tumors

**DOI:** 10.1038/s41417-021-00303-x

**Published:** 2021-02-18

**Authors:** Karan Kohli, Venu G. Pillarisetty, Teresa S. Kim

**Affiliations:** University of Washington, Department of Surgery, Seattle, WA USA

**Keywords:** Chemokines, Tumour immunology

## Abstract

Immune cell infiltration into solid tumors, their movement within the tumor microenvironment (TME), and interaction with other immune cells are controlled by their directed migration towards gradients of chemokines. Dysregulated chemokine signaling in TME favors the growth of tumors, exclusion of effector immune cells, and abundance of immunosuppressive cells. Key chemokines directing the migration of immune cells into tumor tissue have been identified. In this review, we discuss well-studied chemokine receptors that regulate migration of effector and immunosuppressive immune cells in the context of cancer immunology. We discuss preclinical models that have described the role of respective chemokine receptors in immune cell migration into TME and review preclinical and clinical studies that target chemokine signaling as standalone or combination therapies.

## Introduction

The immune system is a dynamic and complex, yet highly organized, network of cells. Immune cells are motile and migrate to specific organs in a context-specific manner. Moreover, they need to come into spatial proximity with other cells to exchange information and function. Diverse immune responses in different situations are a result of these interactions. When they reach their destined organs, immune cells move relatively shorter distances to reach the appropriate microanatomical niche. This intra-organ movement is critical and determinative of immune outcomes [1, 2]. The inter- and intra-organ movement of immune cells is guided by a set of secreted molecules called chemokines. Immune cells that express the cognate chemokine receptor migrate based upon gradients of the respective ligands in a process called chemotaxis. Chemokines were originally discovered as inflammatory cytokines that could guide leukocytes to sites of inflammation, but it is now known that chemokines have additional roles even in the absence of inflammation [3]. For example, chemokines play a major role in the development of lymphoid organs [1, 4]. Defects in the expression of chemokines or chemokine receptors have been associated with dysfunctional lymphoid organ development and defective, aberrant, or exacerbated immune response [4–9]. Thus far, 50 chemokine ligands and 20 chemokine receptors have been described, and all but 6 chemokine receptors respond to multiple chemokines. Chemokines are grouped into four main classes depending on the location of the first two cysteine (C) residues in their protein sequence: namely, CC, CXC, C, and CX3C chemokines. Most chemokine receptors are transmembrane-spanning heterotrimeric G-protein-coupled receptors [1]. Cognate chemokine receptor binding induces G-protein coupling and subsequent activation of downstream signaling proteins involved in cell migration such as Rac, Rho, and Cdc42. The net effect is movement of the cells towards the chemotactic gradient [1].

**Fig. 1:**
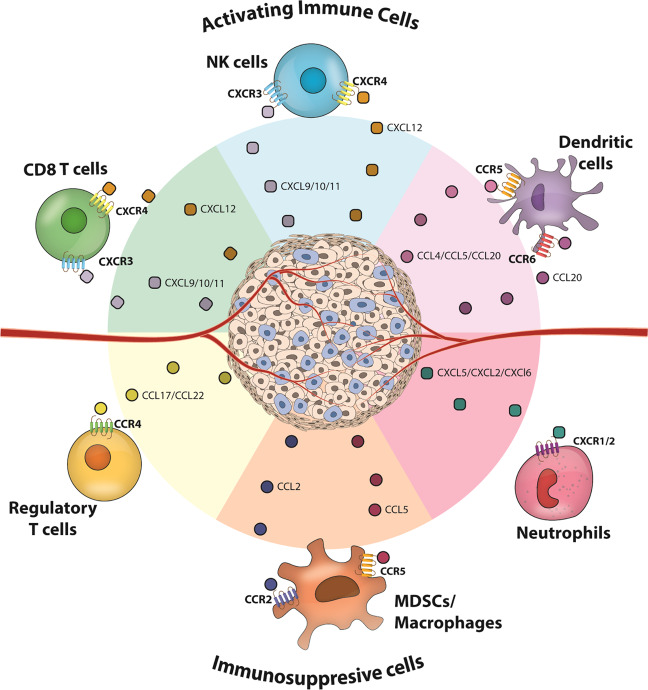
Key chemokines inducing immune infiltration into solid tumors. Illustration depicts chemokines that have been commonly found to guide different types of immune cells into solid TME.

The infiltration of immune cells in the TME is a key factor in cancer prognosis, and chemokines play an essential role in guiding the migration of both activating and suppressive immune cell types [10–13]. The migration of immune cells into tumor tissue is more unpredictable than homeostatic migration of immune cells into lymphoid organs, as solid tumors are ectopic, heterogeneous, and do not have a defined anatomy. Even among tumors of the same type, the migration patterns of immune cells vary by case and time. Nonetheless, understanding the chemotactic environment of solid tumors and identifying chemokines that regulate immune cell entry into solid tumors is imperative in improving current immunotherapeutic interventions, including immune checkpoint blockade (ICB) (Fig. 1). Chemokines also play a role in the intrinsic growth and metastasis of tumor cells, mechanisms of which have been reviewed previously [14]. In the current review, we discuss chemokines that can guide anti-tumor effector and immunosuppressive immune cells into solid tumors and how these chemotactic axes have been targeted to develop immunotherapies.

## Chemokines recruit effector anti-tumor immune cells to the TME

### CXCR3 and CXCR4 direct the migration of T cells and NK cells in solid tumors

Effector immune cells such as activated CD8^+^ T cells and NK cells can recognize and lyse tumor cells [15, 16]. In addition to their cytotoxic function, they secrete the major stimulatory cytokine interferon gamma (IFNγ) to induce and maintain interferon-induced anti-tumor immunity [17]. To mediate anti-tumor effects, effector cells must first enter the tumor by engaging chemokine receptors expressed on their cell surfaces. Lack of intratumoral T cell infiltration is associated with resistance to widely applied therapies including ICB [18]. Upon activation, T cells and NK cells upregulate expression of the chemokine receptor, C–X–C chemokine receptor (CXCR) 3. CXCR3-expressing cells follow gradients of the interferon-inducible ligands, C–X–C chemokine ligand (CXCL) 9 (MIG), CXCL10 (IP-10), and CXCL11 (I-TAC). CXCR3 guides the migration of NK cells into lymph nodes (LNs) and tumors [19, 20]. CXCR3-dependent T cell infiltration has been demonstrated in murine models of lymphoma, renal cell carcinoma, melanoma, and breast cancer [21–25], and is a prerequisite to the success of programmed cell death protein-1 (PD-1)/programmed cell death protein ligand 1 (PD-L1) blockade therapy [26]. In line with the above murine models, increased expression of CXCL9 and CXCL10 has been associated with increased infiltration of activated T cells in many human cancers, including melanoma, ovarian, and colon cancer [27–30]. Indeed, a study correlating chemokine expression and CD8^+^ T cell infiltration in human solid tumors showed that chemokine C–C motif ligand (CCL) 5 and CXCL9 had the highest correlation with CD8^+^ T cell infiltration across different cancer types and also demonstrated cooperation between CXCL9/10 and CCL5 in recruiting effector T cells into tumors [31].

IFNγ is the major driver of CXCR3–CXCL9/10/11 axis. CXCR3 is inducible by IFNγ; CXCL9 is inducible by IFNγ but not by IFNα/β (type-1 interferon); CXCL10 is inducible by both IFNγ and IFNα/β and tumor necrosis factor alpha [32–36]. Indeed, systemic administration of IFNγ increased levels of CXCL10 and intratumoral T cell infiltration in a phase 0 clinical trial for sarcoma, and intratumoral injection of IFNγ increased tumor CXCL10 and CXCL11 in melanoma patients [37, 38]. Strategies to directly increase the level of CXCR3 ligands have been described in preclinical models. These include plasmid-borne CXCL9 [39], intra-tumor injection of CXCL9 [22], recombinant CXCL10 protein with adoptive cell therapy (ACT) [40], intra-tumor injection of CXCL10 [41], retroviral transduction tumor cells with CXCL10 [42, 43], and intraperitoneal injection of oncolytic vaccinia virus expressing CXCL11 [44]. All the above strategies were effective in increasing T cell infiltration and reducing tumor growth in animal models but have not yet been investigated in clinical trials. Several challenges in taking the above therapeutics into the clinic can be envisaged. Injection of naked protein cannot guarantee their lasting bioavailability. Gene therapy can overcome this but would rely on site-specific and high transduction rate within the tumor, which would be difficult to achieve and evaluate.

Alternate interventions including ICB indirectly modulate levels of CXCR3 ligands, presumably through increased production of IFNγ from activated T cells. PD-1 blockade increased tumor expression of CXCL10 in melanoma-bearing mice treated with ACT [45]. Higher levels of CXCL9, CXCL10, and CXCL11 were also detected in melanoma patients’ tumors following treatment with ipilimumab, an antagonistic antibody against cytotoxic T-lymphocyte-associated protein 4 (CTLA4), another inhibitory receptor expressed by T cells [46]. Interleukin-7 increased the expression of CXCL9 and CXCL10 in a murine lung cancer model [47]. Cyclooxygenase-2 (COX-2) is expressed by many cancer types and confers resistance to chemo- and radiotherapy [48]. The COX-2 inhibitor indomethacin enhanced IFNγ-induced expression of CXCL9/10 by ovarian cancer cell lines. In the same study, expression of COX-2 was also shown to be negatively correlated with CXCL10 expression [49]. Indomethacin and acetylsalicylic acid (aspirin), alternative COX inhibitors, similarly enhanced IFN-γ-mediated CXCL9 and CXCL10 in breast cancer cell lines [50]. Celecoxib, another COX-2 inhibitor, together with anti-PD-1 synergistically increased the production of CXCL9/10 [51]. Therapeutics that target toll-like receptors also induced expression of CXCL9-11 at the tumor site [52, 53]. Thus, various immunotherapies may rely on IFNγ-stimulated CXCR3/CXCL9-11 signaling and associated effector cell recruitment for maximal therapeutic effect.

CXCR4 is important for homing of naïve and memory CD8^+^ T cells and NK cells into bone marrow (BM) [54, 55]. CXCR4 also regulates homeostatic proliferation and memory maintenance of T cells, and the development of NK cells [56, 57]. CXCR4 can guide NK cells to tumors. NK cells genetically engineered with a chimeric antigen receptor (CAR) to over-express CXCR4 along with the target antigen receptor demonstrated enhanced mobilization towards CXCL12 (stromal cell-derived factor 1, SDF-1), the exclusive cognate ligand for CXCR4, and increased infiltration in a mouse model of glioblastoma [58]. However, CXCR4 can also prevent adequate infiltration of T cells into tumors, as the surrounding CXCL12-rich stroma can arrest CXCR4-expressing T cells from directly reaching carcinoma cells [59]. Additionally, a phenomenon where high concentration of chemokines repelled cells expressing the cognate receptor was demonstrated for CXCR4/CXCL12 axis in a murine melanoma model. This was termed chemorepulsion or fugetaxis. Tumor expressing high levels of CXCL12 repelled CXCR4-expressing T cells and evaded an anti-tumor response [60].

Pharmacological agents targeting the CXCR4/CXCL12 axis were developed to inhibit tumor growth and metastasis [61] and also mobilize hematopoietic stem cells to the periphery for subsequent transplantation [62]. AMD3100 is a small-molecule CXCR4 antagonist commonly used in the clinic in the latter context [63]. Two independent studies demonstrated that AMD3100 promotes T cell infiltration into the tumor, by releasing them from the CXCL12-rich surrounding stroma [59, 64]. Another CXCR4 antagonist, BL-8040, released T cells from the BM to infiltrate tumors in a mouse lung tumor model [65]. Moreover, in a phase I/II trial of patients with chemotherapy-refractory pancreatic ductal adenocarcinoma (PDA), administration of the CXCR4 inhibitor BL-8040 with a PD-1 blocking antibody led to increased effector T cell infiltration, as well as substantial disease control rates when combined with systemic chemotherapy [66]. Thus, targeting the CXCR4/CXCL12 axis can optimize intratumoral T cell localization, promote T cell anti-tumor activity, and enhance the efficacy of ICB and cytotoxic chemotherapy.

### CCR5 and CCR6 orchestrate dendritic cell migration into tumors for maximal anti-tumor immunity

Antigen-presenting cells (APCs) are crucial to anti-tumor immunity [67]. Dendritic cells (DCs) are the most proficient at antigen presentation, and a particular subset of DCs, i.e., cDC1s, are specialized at cross-priming CD8^+^ T cells and have been shown to play a non-redundant role in eliciting anti-tumor immune responses [68, 69]. Moreover, tumor-infiltrating DCs, especially CD103^+^ DCs, boost the infiltration of effector T cells into the tumor by producing CXCL9/10 and hence recruiting CXCR3^+^ T cells [70]. The mechanisms of intratumoral DC migration, and the chemokines involved, have been less extensively studied in comparison to T cells. However, chemokine receptors involved in DC migration in other disease settings have been described previously [71]. A few reports have implicated the role of CCR5 and CCR6 in the recruitment of DCs into the TME. CCR5 is a key receptor through which HIV enters target cells, but it also mediates physiologic functions of immune cells [72, 73]. CCR5 expression by DCs was required for their response in a parasite infection model [74]. CCR5 was also elevated in DCs in multiple sclerosis and acute monosymptomatic optic neuritis patients [75]. CCR5 expression on DCs has not been clearly demonstrated in tumor models or patients. However, in a genetically engineered mouse model of melanoma, defective expression of CCL4 (macrophage inflammatory protein-1-beta), a ligand for CCR5, led to reduced DC recruitment to tumors [76]. Using the same model, another study showed that CCL5 (RANTES), another ligand for CCR5, was crucial for DC recruitment into tumors. In this study, NK cell infiltration was required for production of CCL5 and subsequent recruitment of DCs [77]. Such NK cell-mediated recruitment of DCs was also demonstrated in a murine colon tumor model [78]. The above two studies suggest that immune cells can sequentially enter tumors and guide the migration of additional incoming cells by specific chemokine secretion.

CCR6 guides DCs into sites of inflammation [79, 80]. The expression of CCL20, a ligand for CCR6, is associated with higher infiltration of immature DCs in breast carcinoma patients [81]. Furumoto et al. [82] showed that CCL20 increased DC infiltration into murine melanomas by using either CCL20-transduced tumor cells or intratumoral CCL20 protein injections.

In the anti-tumor immunity cycle, DCs have to migrate into the tumor, capture tumor antigens, then exit the tumor and migrate to the draining LN to prime naïve tumor-specific T cells [67]. This means that unlike other immune cells, DCs must travel into and egress out of the tumor. Activation-induced CCR7 expression on DCs is crucial for their migration from peripheral tissues into the LN following the gradients of cognate ligands CCL19/21 [5]. The level of CCR7 expression correlates with their capacity to migrate into LNs and also from the LN periphery to the deep T cell zone of the LN [83]. It is reasonable to hypothesize that once DCs are in the tumor parenchyma, they use the same CCR7-dependent migration to make their way into the draining LN; however, this has not been demonstrated. Whether intratumoral DCs can access terminal lymphatics in tumors for their CCR7-dependent migration towards the draining LNs needs remains to be investigated. DCs are crucial to anti-tumor immunity, but much works remains to be done to identify the dominant chemokine(s) affecting their migration to and from the TME.

## Chemokines recruit immunosuppressive cells into the TME

### CCR4 regulates migration of regulatory T cells into TME

Regulatory T cells (Tregs) are a specialized subset of CD4+ T cells that are highly immunosuppressive and play a crucial role in maintaining immune tolerance during homeostasis and suppressing exacerbated immune responses in various pathological conditions [84]. In cancer models, they have been shown to suppress the anti-tumor immune response and promote tumor growth [85]. CCR4 expression has consistently been shown to mediate Treg migration in various cancer models. Primary tumor cells and tumor-associated macrophages (TAMs) from ovarian tumors produced CCL22, a ligand for CCR4, which was crucial for the migration Tregs, whose CCR4 expression was higher than other CD4+ T cells [86]. The group of Christine Ménétrier-Caux in two different studies demonstrated CCL22-mediated recruitment of Tregs in breast cancer [87, 88]. Elevated expression CCL22 and CCL17, an alternate ligand for CCR4, was associated with higher Treg infiltration in gastric cancer and esophageal squamous cell carcinoma [89, 90].

Several strategies to abrogate CCR4–CCL22/17 axis-mediated Treg recruitment, including blocking antibodies, siRNAs, and antagonists, have been effective in preclinical cancer models [91–94]. Many therapeutics targeting this axis are currently being investigated in clinical trials. For example, mogamulizumab (KW-0761) is a humanized, glycoengineered IgG1κ monoclonal antibody that targets CCR4-expressing cells by antibody-dependent, cell-mediated cytotoxicity (ADCC) [95]. Although it was originally developed to target malignant T cells in lymphomas and leukemias, clinical trial results show that it abrogates Treg accumulation in lymphomas [96, 97]. Objective responses were noted for 13 of 37 lymphoma patients (35%; 95% CI, 20–53%), including five patients (14%) with complete response; however, it is difficult to ascertain whether the effect was caused by depletion of Tregs or antibody-dependent cellular toxicity to CCR4-expressing T cell lymphomas [97]. A clinical trial of mogamulizumab in lung and esophageal cancer patients demonstrated that the antibody was well tolerated and led to efficient depletion of Tregs [98]. Allosteric antagonists that bind to the intracellular domain of CCR4 (class II antagonists) have been developed by pharmaceutical companies, including GlaxoSmithKline (GSK2239633) and AstraZeneca (AZD-1678, AZD-2098) [99, 100]. RAPT pharmaceuticals have developed small-molecule inhibitors of CCR4, including FLX475 and RPT193. Phase 1/2 trials with FLX475 in combination with pembrolizumab for advanced or metastatic cancer have been recently completed but not yet reported (NCT03674567). The full therapeutic potential of CCR4 inhibition and Treg depletion remains to be defined.

### CCR2 and CCR5 regulate the migration of tumor-associated macrophages and myeloid-derived suppressor cells

Monocytes are pliable cells that develop in the BM but can further differentiate in peripheral tissues. Depending on the environment, they differentiate into immunostimulatory or immunosuppressive cells [101]. Monocytes bear resemblance to DC-precursors in ontogeny and can function as inflammatory APCs. However, in the context of cancer, monocyte-derived TAMs have frequently been shown to contribute to tumor progression and are associated with poor clinical prognosis [102, 103]. Myeloid-derived suppressor cells (MDSCs) are closely related to monocytes but are believed to arise from precursors distinct from monocytes [104, 105]. Owing to their close resemblance with monocyte-derived macrophages, MDSCs are often described interchangeably with TAMs, especially in the context of human tumors, and have similar immunosuppressive functions. In homeostatic conditions, CCR2 is exclusively expressed by myeloid cells, especially monocytes, and guides migration from BM to peripheral sites during both homeostatic and inflammatory conditions [106]. CCR2 is pivotal for the migration of monocytes and MDSCs into solid tumors [107]. Tumors in *Ccr2* knockout mice are minimally infiltrated by MDSCs, which results in reduced tumor growth and metastasis [108–110]. Once myeloid cells infiltrate the tumor, they can further produce the cognate ligand CCL2 and maintain or even augment monocyte trafficking into tumors [111, 112]. Generally, tumor cells and tumor-associated stroma are rich sources of CCL2, the ligand for CCR2 [113, 114]. Elevated CCL2 expression in tumors was shown to recruit high numbers of monocyte or monocyte-derived cells in mouse models of glioma, renal tumors, lung cancer, prostate cancer, and melanoma [115–120]. High levels of CCL2 correlated with increased monocyte/macrophage recruitment and was an indicator of adverse prognosis in patients with breast, ovarian, gastric, and esophageal carcinomas [108, 121–124]. A study compared the levels of CCL2 in tumor tissue and adjacent healthy tissues from patients with breast, gastric, and ovarian cancer, and consistently observed an increased level of CCL2 in tumor samples [125].

Therapeutics that disrupt the CCR2/CCL2 axis have been effective in blocking macrophage infiltration and reducing tumor growth and metastasis in preclinical models of cancer, with variable activity demonstrated in early phase clinical trials. The therapeutic benefit of anti-CCL2 antibodies was seen in a mouse model of renal cell carcinoma [126]. Inhibition of CCR2/CCL2 with a CCL2 blocking antibody inhibited the infiltration of monocytes and metastatic seeding of breast cancer cells in mice [127]. In a phase II clinical trial for patients with cancer metastatic to the bone, an anti-CCR2 antibody (MLN1202) caused considerable reduction in urinary N-telopeptide (uNTX) values after 43 days of treatment in for 14 out of 43 patients with bone metastasis [128] (ClinicalTrials.gov ID: NCT01015560). A CCR2 inhibitor (PF-04136309) reduced TAM infiltration and tumor growth in a syngeneic PDA tumor mouse model [129]. A phase 1b trail of PF-04136309 in combination with nab-paclitaxel/gemcitabine in metastatic PDA patients demonstrated a decrease in inflammatory monocytes (IM) in the peripheral blood, without accumulation in the bone marrow. Unfortunately, CCR2 inhibition also caused increased pulmonary toxicity compared with standard chemotherapy [130]. In another phase 1b trial, a combination of PF-04136309 and FOLFIRINOX, a different standard chemotherapy regimen, was safe, reduced monocyte egress from BM, TAM infiltrate in the primary tumor, and resulted in a higher than expected objective response rate (49%) in patients with borderline resectable and locally advanced pancreatic cancer [131]. Another CCR2 antagonist, CCX872, in combination with FOLFIRINOX was safe and increased overall survival compared to FOLFIRINOX alone (29% versus 18.6% at 18 months) in pancreatic cancer patients [132]. CCR2 is the most common target of therapeutics developed to block monocyte infiltration into tumors.

Although the CCR2/CCL2 axis has been consistently found to mediate the recruitment of TAMs/MDSCs, other chemokines and receptors have also been shown to contribute to the process. In a transgenic mouse melanoma model, CCR5-expressing MDSCs induced immunosuppression in tumors. The same study also showed an enrichment of CCR5^+^ MDSCs and increased concentration of the cognate ligands, i.e., CCL3, CCL4, and CCL5, in melanoma specimens compared to matched serum [133]. In mice injected with TRAMP prostate cancer cell line, CCR5 guided CD11b^+^Ly6G^+^Ly6C^low^ polymorphonuclear granulocytic MDSCs from BM to tumors where they induced immunosuppression. The cognate ligands produced in the tumor, i.e., CCL3/4/5, not only recruited MDSCs but also induced the proliferation of MDSC-progenitors in the BM [134]. In the above two studies, it was shown that MDSCs that expressed CCR5 were more immunosuppressive than their CCR5^−^ counterparts. In a mouse model of Her2-driven breast cancer, CCL5 over-expression promoted tumor recurrence by recruiting CCR5-expressing macrophages [135]. Elevated CCL5 expression is also associated with disease progression in breast [136], ovarian [137], gastric [138], and pancreatic cancer [139]. It remains to be determined what contextual factors drive CCR5-mediated suppressive versus stimulatory myeloid infiltration into the TME.

Inhibition of the CCR5 axis has been tested in preclinical models and early phase clinical trials. Maraviroc, a CCR5 antagonist, repolarized macrophages to an immune-active phenotype in organotypic explants derived from patients with metastatic colorectal cancer (CRC). In the same study, it was reported that a clinical trial of Maraviroc in patients with metastatic CRC demonstrated partial responses in patients with previously treatment-refractory disease. The effects included marked reductions in key cytokines and growth factors promoting tumor growth, chemotherapy resistance, or angiogenesis [140]. CCR5 expression on many different types of cancer cells promotes metastatic phenotype, metabolic and cell survival pathways, angiogenesis, and DNA repair [73]. Thus, CCR5 antagonists, developed for HIV treatment, have also been tested as cancer therapeutics. Some of these antagonists could inhibit tumor growth through dual mechanisms, i.e., inhibiting intrinsic tumor cell activity and reducing suppressive macrophage infiltration in the tumor. For example, Met-CCL5, an antagonist of CCR5, reduced the frequency of infiltrating macrophages in a murine model of breast cancer [141]. mCCR5–Ig fusion antibody, which sequesters CCR5 ligands, reduced MDSCs and tumor growth in a transgenic melanoma model [133]. Maraviroc inhibited TAM infiltration and tumor growth in mice inoculated with BM1 tumor cells [142]. Because CCR5 expression can guide both immunosuppressive or immune-stimulatory myeloid cells, its effects on both should be carefully considered before targeting this receptor.

### CXCR1 and CXCR2 direct migration of neutrophils

Neutrophils, immune cells of the myeloid lineage, act as first responders to inflammation. At inflammatory sites, neutrophils play a role in phagocytosis, intracellular lysis of pathogens, and wound healing. Neutrophil infiltration has been observed in many cancer types and implicated in both anti- and pro-tumor roles [143]. Tumor-associated neutrophil have been generally classified into “N1” versus “N2” depending on if they exhibit anti-tumor or pro-tumor features respectively [144]. However the consensus leans towards the pro-tumorigenic role of neutrophils and many lines of evidence show neutrophils contribute to neovascularization in tumors, much akin to their role in wound healing, and thus promote tumor growth and metastasis [145]. Neutrophils express CXCR1 and CXCR2, which guide them towards their dominant ligand, CXCL8, as well as alternate ligands CXCL1, CXCL2, CXCL5, and CXCL6 [146]. CXCL8 expression is elevated in many cancers, including melanoma, colon, lung, prostate, and ovarian carcinoma [146]. CXCL5 and CXCL2 levels were high in tumor cells and tumor-associated stroma in a mouse model of PDA. Genetic ablation of CXCR2 resulted in lower neutrophil infiltration and suppression of tumor growth [147]. CXCR1–CXCL8 axis was essential in recruiting neutrophils to the tumor site in a zebrafish model of glioblastoma [148]. CXCL1 and CXCL2 chemokine gradients in melanoma-bearing mice induced neutrophil recruitment and tumor angiogenesis [149]. Hepatocellular carcinomas (HCC) have high expression of CXCL5, which attracts neutrophils, and is an indicator of poor prognosis [150]. CXCL6 over-expression in a mouse model of melanoma resulted in high neutrophil infiltration and increased angiogenesis [151]. Even in reports where neutrophils have been shown to exert an anti-tumor effect, CXCL5 and CXCL8 were shown to positively correlate with neutrophil infiltration [152].

Therapeutic agents that disrupt CXCR1/CXCR2 receptor binding with cognate ligands have been efficacious in suppressing tumor growth as shown in preclinical studies. Reparixin, a clinical grade CXCR1/2 inhibitor, suppresses breast cancer growth in vitro [153]. Small-molecule antagonists of CXCR1/2, including SCH479833 and SCH527123, exerted anti-tumor activity in xenograft models of breast cancer, CRC, spontaneous colon cancer liver metastasis, and melanoma [154–157]. ABX-CXCL8, a CXCL8 blocking antibody, inhibited melanoma growth in a xenograft model [157]. However, in the above studies the effect of therapeutics on migration of neutrophils was not demonstrated and remains to be evaluated in the clinical setting.

## Concluding remarks

The bioavailability of chemokines in a solid tumor microenvironment critically influences the immune cell composition therein. However, research to understand the regulation of chemokine expression in tumors can be challenging due to heterogeneity of solid tumors and time-dependent changes in chemokine expression. More progress has been made on our understanding of the relevant chemokine receptors guiding immune cell migration into the TME. Although multiple chemokine receptors have been implicated in the migration of each immune cell type into the TME, one or two dominant receptors for each cell type have been consistently found to play a role for each cell type across various preclinical models (Table 1). While some strategies targeting these receptors have shown preliminary success in clinical trials, either alone or in combination with other interventions such as ICB and ACT, many strategies effective in mouse models, e.g., intratumoral injection of CXCR3 ligands, remain to be studied in patients. Therapies that block chemokine signaling could be easier to develop and monitor than strategies to constitutively increase chemokine expression in tumors. Pharmacological agents developed for other purposes have also been shown to manipulate immune cell trafficking into cancer. For example, antagonists to CXCR4, CCR2, CCR5, and CXCR1/2 were developed to disrupt tumor invasiveness, but now have been observed to influence immune cell migration in the TME. Targeting chemokines and receptors to improve cancer treatment can also be challenging, as expression is not exclusive to one immune cell type, and thus inhibiting one could have pleiotropic effects on several types of immune cells. Thus, the selection of candidate chemokine axes to target requires consideration of collateral effects. Biomarker assays to measure chemokine levels, receptor expression on cancer cells and immune cells, and immune cell infiltrate will be necessary to identify ideal pathways to target in an individual patient’s tumor. Understanding and manipulating intratumoral chemokine signaling offers a promising avenue to improve responses to current immunotherapies.

**Table 1:** Chemokines axes regulating migration of immune cells in cancer.

Cell type	Chemokine axis	Role implicated in tumor type	Study species
CD8^+^ T cell	CXCR3–CXCL9/10/11	Lymphoma [21], melanoma [24], CRC [26]	Mouse
		Metastatic melanoma [27], epithelial ovarian cancer [30, 31]	Human
	CXCR4–CXCL12	PDA [64]	Mouse
		PDA [59]	Human
NK cells	CXCR3–CXCL9/10/11	Melanoma [158, 159], lymphoma [19]	Mouse
	CXCR4/CXCL12	Glioblastoma [58]	Mouse
DCs	CCR5–CCL4/CCL5/CCL20	Melanoma [76, 77],	Mouse
	CCR6–CCL20	Breast carcinoma [81]	Human
Tregs	CCR4–CCL17/CCL22	Ovarian tumors [86]	Mouse
		Breast tumors [87], esophageal squamous cell carcinoma (ESCC) [90]	Human
MDSCs/TAMs	CCR2–CCL2	ESCC [108], lymphoma [110], glioma [115], renal tumor [116], lung cancer [117], prostate cancer [119]	Mouse
		ESCC [108], glioma [115]	Human
	CCR5–CCL5	Melanoma [133], prostate cancer [134], breast cancer [135]	Mouse
		CRC [140]	Human
Neutrophils	CXCR1/2–CXCL5/CXCL2/CXCl6	PDA [147], melanoma [149, 151], HCC [150]	Mouse
		Glioblastoma [148]	Zebrafish
		HCC [150]	Human
